# Cardiopulmonary Bypass: a Forgotten Area of Searching for New Knowledge in Brazil and the Importance of Translational Research

**DOI:** 10.5935/1678-9741.20160073

**Published:** 2016

**Authors:** Rodolfo Neirotti

**Affiliations:** 1 Clinical Professor of Surgery and Pediatrics, Emeritus Michigan State, Brookline, MA, USA and honorary member of Sociedade Brasileira de Cirurgia Cardiovascular (Brazilian Society of Cardiovascular Surgery), São Paulo, SP, Brazil.

The first heart transplantation in Latin America was performed less than six months after the pioneering work of Christian Barnard in this field^[1]^. As impressive as the fact that this procedure was the 17^th^ heart transplant done in the world, is that this surgery was performed with a cardiopulmonary (CPB) machine made in Brazil.

At that time, the homegrown cardiac surgery was side by side with the cardiac surgery of the developed world due to the capabilities of the bioengineering industry and the remarkable surgical skills and dedication of the local pioneers^[2]^. However, nowadays, there is an industry stagnation with low technology incorporation in the country and a gap in this field with the international technological development due to multiple factors. The diminished number of publications by Brazilian authors - less than 0.9% of total worldwide publications - registered in PubMed (US National Library of Medicine National Institutes of Health) database ratify the scientific stagnation in the knowledge of CPB.

Translational research, a "bench-to-bedside and beyond" approach, aims to improve individual and public health by generating multicenter and multidisciplinary collaboration to pull discoveries from basic science arising from laboratory, clinical, or population studies into clinical applications^[3]^.

## The importance of testing the devices used in our clinical practice

Recently, driven by the need of introducing translational research in CPB, the Heart Institute of University of São Paulo (Instituto do Coração da Universidade de São Paulo) began collaborative studies in pediatric CPB with the Penn State University Health Center for Pediatric Cardiovascular Research.

Brazil has a large number of medical devices manufactured and available only in this region that are approved by the National Health Surveillance Agency (Agência Nacional de Vigilância Sanitária - ANVISA) without further clinical data or benchmarking with other similar devices. Moreover, only FDA approved or widespread used products in the developed countries are object of research by the international scientific community.

Monitoring pressure, blood and water temperature, use of bubble detectors and blood level sensors devices are not incorporated neither in the clinical perfusion guidelines, nor in the training school curriculum and clinical practice. Furthermore, despite that some imported CPB machines equipped with all these safety devices are available for use in a great number of cardiac centers, only less than 5% of them are used with the servo control mode turned on. In other words, the majority of the clinical perfusions are conducted without any automatic safety device control, thus making impossible to identify, explain and document undesirable events. Building a stronger knowledge about CPB through basic research and clinical trials, and the incorporation of the new information in the perfusion curriculum, will help the management of children with congenital heart defects (CHD).

Altogether, a better understanding of the hemodynamics characteristics and differences among these devices, as well the interaction between them and the patients, will help to improve clinical outcomes in pediatric cardiovascular surgery in Brazil and other Latin American countries using them.

The manuscript entitled "*In-vitro* evaluation of two types of neonatal oxygenators in handling gaseous microemboli and maintaining optimal hemodynamic stability during cardiopulmonary bypass", published as an original article in this issue of the Brazilian Journal of Cardiovascular Surgery, represents an important initiative of an international multicenter and multidisciplinary collaboration. This study was conducted at the Pediatric Cardiovascular Research Center at the Penn State Milton S. Hershey Medical Center, under the supervision and mentorship of Prof. Akif Undar^[4]^. The purpose of this joint venture was to understand the characteristics of a Brazilian oxygenator by using an internationally used oxygenator as a benchmark rather than to determine the superiority of one of them.

READ ARTICLE ON PAGE 343

It is well known that only FDA approved devices are used in large clinical trials published by the international scientific community. Thus, this study represents the first published evaluation of the hemodynamics and handling capabilities of microemboli in a Brazilian made oxygenator, using a FDA approved oxygenator as a point of reference. Both oxygenators are clinically used for the same population and although with different characteristics regarding maximum flow and prime volume, they have a very similar hemodynamic performance. The undesirable passage of microemboli from the venous side of the oxygenator to the patient is sometimes forgotten, as well that the capability of capturing them by the oxygenator may be different and dependent on the membrane area/maximum flow rate ratio.

Clinically, rather than the existence of microemboli in the arterial side of the oxygenator, the most important issue is to diminish the passage of air to the patient by placing an arterial filter in the circuit. Unfortunately, this practice is not yet part of the routine.

I am aware that another study was done recently, in order to further understand the role of an arterial filter - to be published soon - testing the Braile pediatric oxygenator with and without an arterial filter in the CPB circuit, using another FDA approved oxygenator as a benchmark.

The knowledge acquired with these and other experiments in CPB should enable the authors and others to improve the outcomes for their CHD patients. They should be congratulated for their initiative and efforts to embrace international parameters in their clinical practice.
